# Study of trioleoylglycerol two-layer and adiposome cross-section mimicking four-layer systems through atomic-level simulations

**DOI:** 10.1063/4.0000168

**Published:** 2022-12-05

**Authors:** Ahmed Hammad Mirza

**Affiliations:** Department of Biosciences, COMSATS University Islamabad, Sahiwal Campus, Sahiwal 57000, Pakistan

## Abstract

Adiposomes are artificially prepared lipid droplet (LD)-mimetic structures, which, unlike LDs, do not harbor proteins. The dynamics of interaction between triacylglycerols (TAGs), drug molecule, and phospholipids in adiposomes is currently not well-established. Trioleoylglycerol (TOG) molecule was divided into three parts: two oleoyl tails and one 2-monooleoylglycerol (MOG). Forcefield parameters for two oleoyl tails were adopted from the AMBER18 repository while that of the MOG forcefield was taken from the literature. Charge correction was performed on the MOG forcefield before its utilization. After charge correction, the resulting TOG molecule had zero charge. TOG bilayer (2L) and tetralayer (4L) systems were prepared and simulated. TOG bilayer (2L) systems—modeled from two different initial conformations, the TOG3 conformation and the TOG2:1 conformation—showed that TOG2:1 conformation was more prevailing irrespective of the starting conformation and was subsequently used in further simulations. The hydrated TOG 2L system showed TOG–water solution solubility of 0.051 mol L^−1^ which is near experimental values. This validated the correct parameterization of the TOG molecule. The simulations of 4L systems showed stable membrane behaviors toward the end of simulations. It was also observed that in the 4L system, the TOG molecules showed the formation of micelles with the drug molecule. Almost six TOGs remained continuously in contact with the drug molecule throughout the simulation. The availability of charge-corrected TOG parameterization is expected to equip future studies with a framework for molecular dynamics simulations of adiposomes and/or LDs at the atomic level.

## INTRODUCTION

Adiposomes are created with a neutral lipid core containing mostly triacylglycerols (TAGs) covered by a phospholipid monolayer. Adiposomes provide a simplified structural model of fat storage compartments of cells, known as natural lipid droplets (LDs).^1^ In addition, natural LDs serve as a platform for the function of a class of specialized proteins, whereas adiposomes are usually constructed without these proteins.^2^ The study of LDs, and, thus, that of LD-mimetic adiposomes, sometimes also referred to as artificial lipid droplets, is important due to their function in lipid homeostasis and related involvement in different diseases;^3,4^ however, adiposomes could also serve as an efficient pharmaceutical drug carrier system.^5,6^ Currently, in the LD biology field, the key questions are how TAGs are organized within the phospholipid monolayer and what is the basis of specific LD-binding of proteins to adiposomes/LDs (A/LD), both of which are directly related to the structure of the phospholipid–TAG interface. Even the dynamics of drug–TAG interactions within an A/LD are terra incognita in the LD biology field. However, due to the subtle and peculiar nature of A/LDs, current state-of-the-art experimental methods are unable to provide information about molecular packing with high enough resolution. Therefore, only computer simulation techniques may provide the means to predict the molecular structure of the A/LD^7^ and may provide essential details. There are mainly four types of simulation techniques: (i) quantum mechanical level (QM), (ii) atomic/molecular level, (iii) coarse-grained (CG) level, and (iv) continuum level (Fig. S1, Ref. 72). Briefly, QM simulations are relatively more accurate and performed at the sub-atomic level. Atomic/molecular level simulations have mostly timescales starting from femto- to nanosecond. CG-type simulations have higher timescales up to micro-/milliseconds, in some cases even more. Continuum simulations are performed at much higher timescales and exercised for bigger system studies.

Due to limitations of computing cost and the availability of required parameters, only a few computer simulation studies have been performed to learn the structural and dynamic properties of A/LDs and apolipoprotein particles. These studies have been proven effective by providing results comparable to experimental outcomes. Examples include modeling high-density lipoprotein particles with cholesteryl esters^8–11^ and oil/water modeling to represent lipid emulsions.^12,13^ Many other aspects of A/LDs are also studied to understand the dynamic behavior of lipids, i.e., from vesiculation of adiposomes^14^ to cholesterol–TAG interactions^15,16^ and interaction of proteins with LDs in some cases.^17–20^

The structure of TAG molecules inside an A/LD is also of equal importance.^21^ There could be two dominant conformations of TAGs to be used for the simulation of an A/LD: the TAG3 type conformation, in which all lipid tails align in the same direction, and the TAG2:1 type conformation, in which two lipid tails point in one direction and the third to the opposite. Yet, it is not well-known which conformation of TAG to use as the initial structure for simulation purposes. However, experimental evidence suggests that TAGs can form lamellar phases with the individual molecules in TAG2:1 conformation.^22,23^ Furthermore, according to available crystal structures, TAGs make lamellar phases where the most probable conformation is TAG2:1.^24–26^ It is unclear, however, whether this conformation of TAG is stable in LDs under provided constraints of the system and the higher (physiological) temperatures. When seeking answers to such questions, atomic-level studies of TAGs are indispensable due to their ability to provide details with higher resolution and conform to actual experiments. One of the impediments of atomic-level modeling studies was the absence of atomic-level parameters for TAG. The basic workflow of atomic-level simulation techniques is to calculate the energies of individual elements of a system based on their defined forcefields.^27,28^ The forcefields include atoms, bonds, charges, angles, and dihedral information of each element of the system, and most importantly, they are calculated through QM technique. The resulting energies are then integrated with defined equations to calculate forces. So far, only two atomic-level forcefields for neutral lipid trioleoylglycerol (TOG) are reported in the literature, one without QM calculations^29^ and the other with QM calculations.^30^

For the current study, the TOG forcefield calculated through QM technique was used.^30^ Before using the forcefield, first, a charge correction was made such that the TOG molecule as a whole had zero charge. Atomic-level simulations were performed for TOG 2L membranes with two dominant conformations, a hydrated TOG 2L system to calculate TOG–water solubility, and an adiposome cross section-mimetic 4-layer (4L) system where the TOG bilayer is sandwiched between DOPC monolayers.

## COMPUTATIONAL METHODS

### MOG forcefield calculation and preparation of TOG molecule

Advanced Model Building with Energy Refinement (AMBER) package uses a modular approach to build lipid molecules so that it has separate forcefields for oleoyl (OL) tails and the glycerophospholipid headgroups, for example, phosphatidylcholine (PC).^31^ TOG molecule was divided into three fragments, two OL tails and one 2-monooleoylglycerol (MOG). Forcefield parameters for two OL tails were adopted directly from the AMBER18 repository. The MOG forcefield parameters obtained from the previous study^30^ were employed in this study. The forcefield parameters for the MOG were calculated through a quantum mechanical (QM) approach using the Restrained Electrostatic Potential (RESP)^32^ and the RESP charge fitting tool through the R.E.D webserver (https://upjv.q4md-forcefieldtools.org/REDServer-Development). The QM calculated glycerol to one lipid tail attached at C2 position interatomic distances, bond angles, and torsion angle parameters are in good agreement with Refs. 23, 33, and 34 and the value of these parameters falls in the range provided in Ref. 35.

The MOG molecule was processed in the xleap module through “edit” command of AMBER.^36^ Element and atom names were made compatible with lipid17 forcefield providing a look that MOG is a lipid molecule. To make space available for attachment of OL tails, the corresponding hydrogens from formyl moieties of MOG (C11 and C31) were removed. The gained charge of two hydrogen-less MOG (hMOG) molecule was corrected utilizing xleap before further processing. The library file (.lib) of hMOG, including forcefield parameters, was saved after several minimization steps. It was made sure that after charge correction, the hMOG molecule had zero charge. When loaded in the AMBER environment, the .lib file of hMOG automatically parameterized all the atoms of the TOG molecule except OL tails. The OL tails took parameterization through the lipid17 forcefield. As a whole, the TOG molecule with hybrid forcefield parameters was prepared. Due to the charge-correction of hMOG, when OL tails were attached to it, the complete TOG molecule had zero charge.

### Preparation of TOG membranes

Based on the available literature, two conformations for TOG molecule were created: (i) TOG2:1 with two lipid tails in one direction and one lipid tail in the opposite direction [Fig. S2(A), Ref. 72] and (ii) TOG3 with all lipid tails in one direction [Fig. S2(B), Ref. 72]. The TOG3-type membranes were prepared using memgen^37^ and AMBAT^38^ tools. As per crystal structure information, the membrane structure for TOG2:1 conformation should be in the form of intercalating layers, whereas memgen and AMBAT tools do not provide such functionality. Therefore, membranes for the former type of conformation were prepared semi-manually by UCSF Chimera.^39,40^ The supplementary information (Data S1, Ref. 72) contains the hMOG library and TOG2:1 and TOG3 PDB formatted files for preparing membranes. TOG bilayer systems, from here on 2L, were optimized with the AMBAT tool of AMBER with TOG atom information. For the adiposome-mimetic (4L) system, the DOPC bilayer in the water environment was prepared through the AMBAT tool. The TOG membrane was then placed between the DOPC leaflets. As provided in the previous study,^30^ similar TOG–TOG distances in 4L systems were used.

### Simulation parameterization and simulation run

The TOG3 2L system and TOG2:1 2L system were first energy minimized and then simulated at 298 K. Slow heating was applied to achieve stable systems at this temperature so that the systems were heated from 0 to 100 K, 100 to 200 K, and 200 K to the final temperature [Table I:E1 and Table S1:E1 (Ref. 72)]. For initial simulations, production molecular dynamics (PMD) was performed for approximately 210 ns for each membrane system under constant pressure (NPT) ensemble with periodic boundary conditions. As in a system, all TOGs were similar, isotropic NPT conditions were applied using Monte Carlo barostat. For the TOG–water system, the simulation was run up to 265 ns [Table I:E2 and Table S1:E2 (Ref. 72)]. Three 4L systems were prepared: (i) with no explicit charges (NC), (ii) with explicit charges (CH), and (iii) a drug molecule inside the core of 4L with explicit charges (DM) and put on simulation with parameters described in Table I:E3 and Table S1:E3 (Ref. 72). It was observed that the DOPC–TOG system cannot be simulated under anisotropic conditions because DOPC membrane deformations were recorded after some steps of production MD. To cope with this problem, the 4L system was simulated under semi-isotropic conditions that seemed stable. For all 4L systems, after minimization, slow heating (as described above for the 2L system) was performed up to 273 K. The slow heating strategy helped avoid membrane deformations due to sudden temperature changes. The heating was first applied to the water molecules while all other atoms were put on restraints. Gradually restraints were relaxed, modeling a process where heat flows from water to the core of the 4L system.

All TOG 2L simulations were conducted in the way that after every 100 ns run on pmemd.cuda,^41^ a 1 ns simulation was run using the sander.MPI module of AMBER.^42^ For the 4L system, after every 20 or 40 ns simulation on pmemd.cuda, a 0.5 ns run was performed on the sander.MPI. This approach helped minimize the probability of local energy minima traps because, with every new simulation, the random seed was provided for the calculations.^43,44^ All the analyses were performed in cpptraj and compiled in ipython modules of AMBER.^45,46^ Graphs were made with xmgrace^47^ and Microsoft Excel.

## RESULTS

TOG forcefield adopted from Ref. 30 was used in the current study, and charge correction was performed by AMBER's built-in modules. With the help of AMBER's built-in commands, it was made sure that after the parameterization of TOG molecules, the overall charge of TOG was zero. The parametrized TOGs were then used to build TOG 2L and DOPC-TOG 4L systems. The details of all simulation experiments and their corresponding parametric information are provided in Tables I and S1 (Ref. 72), respectively.

### Charge-corrected simulation of TOG 2L system showed a similar behavior with charged TOG 2L system

**FIG. 1.**
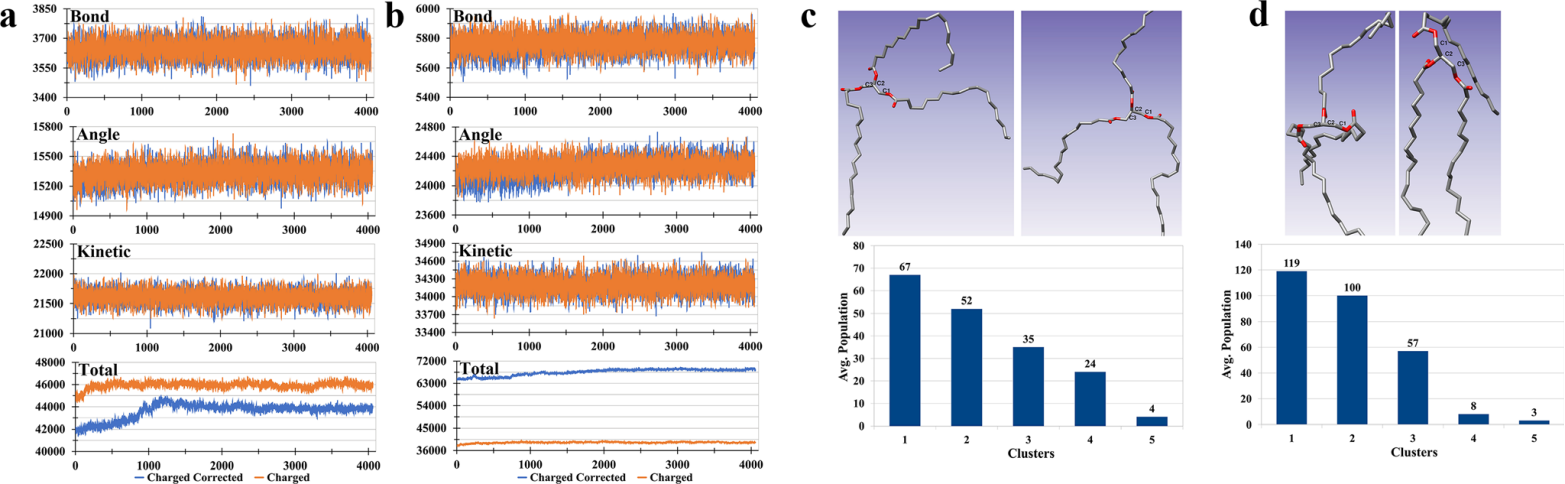
Simulation of charge-corrected 2L system in TOG 2:1 and TOG3 conformations and their comparison to corresponding charged 2L system simulations. The simulation of TOGs only charge-corrected 2L systems were carried out with two dominant TOG conformations and results were compared with charged 2L system simulations. The simulations were executed with almost exact parameters files. The comparison of energy terms including bond energy, angle energy, kinetic energy, and total energy between charge-corrected and charged TOG 2L systems were conducted for TOG 2:1 conformation (a) and TOG3 conformation (b). It was observed that bond energy, angle energy, and kinetic energy for both of the systems showed similar values while total energy of the systems showed distinct values. The TOG conformation analysis with kmeans clustering displayed TOG2:1 conformation as the most popular conformation in TOG2:1 (c) and TOG3 (d) 2L simulations. The values for clusters are shown at lower part and the two most popular structures are shown at upper part.

A total of two 2L systems with charge-corrected TOGs were prepared in two dominant conformations, i.e., TOG2:1 and TOG3 conformations. For comparison purposes to the previous study, the simulations were performed at 298 K with parameters similar to their corresponding charged 2 L systems [Table I:E1 and Table S1:E1 (Ref. 72)]. A comparison was made for the results of charge-corrected simulations with their corresponding charged simulations at 298 K (Ref. 30) (original data were taken by the consent of the corresponding author). An analysis of energy terms demonstrated that the charge-corrected simulations showed almost similar behavior when compared to the charged system. For example, bond energy, angle energy, and kinetic energy for the charge-corrected and charged TOG2:1 system had the same values [Fig. 1(a)]. Only the total energy values showed a difference between both systems. The difference in total energy values was possibly due to electrostatic differences produced due to a small charge of (−0.14) on charged TOGs [Figs. S3(A) and S3(B), Ref. 72]. Almost exactly, similitude behavior was observed for charge-corrected and charged TOG3 2L systems [Fig. 1(b)]. The kmeans clustering with default parameters was performed for conformation analysis of both charge-corrected systems. The results showed that the TOG2:1 conformation was the most populated conformation in the TOG2:1 2L system [Fig. 1(c)] and the TOG3 2L system [Fig. 1(d)]. The two most populated conformations are shown in the figure [Fig. 1(c), upper; Fig. 1(d), upper]. The average population of each conformational cluster from the kmeans algorithm is provided in Fig. 1(c), lower and Fig. 1(d), lower for TOG2:1 and TOG3 2L systems, respectively. This behavior demonstrated that TOG2:1 might be the natural conformation for TOGs and TOG2:1 conformation is the preferred one for simulation systems using TOGs.

**FIG. 2.**
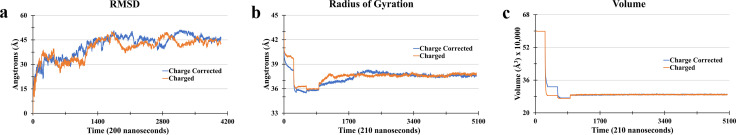
Comparison of structural changes between charge-corrected and charged system from TOG 2L system. An RMSD (a) analysis between charge-corrected and charged TOG2:1 2L systems was performed. It was observed that except with few structural changes, both the systems showed an overall similitude behavior for RMSD. Even the radius of gyration (b) and the volume (c) of both charge-corrected and charged systems showed almost same behavior.

Further analyses were performed for the TOG2:1 2L system, and the results were compared with the corresponding charged 2L system. As with the comparison of energy terms and conformational analysis, the root mean square deviation (RMSD), radius of gyration, and volume analysis also showed similar outcomes between charge-corrected^30^ and charged 2L systems. It was observed that the first peak of RMSD for both systems was at ∼10 Å and behaved almost in a similar fashion afterward [Fig. 2(a)]. Such observation demonstrates that both systems had comparable structural changes during the simulation run. The radius of gyration [Fig. 2(b)] and volume of the system [Fig. 2(c)] showed some differences initially but later on showed similar types of changes toward the end of the simulation. These results suggested that the simulations performed in the previous study with charged 2L systems^30^ were well-performed; in addition, their results are comparable to the charge-corrected 2L systems. It also suggested that the results of other charged 2L systems, including the number of TOGs and ensemble parameterization, can be used in further experiments.

### TOG 2L–water simulation

Formally trioleoylglycerol (TOG) has net zero charge with high solubility in non-polar solvents, for example, chloroform and ether, and is slightly soluble in alcohol. TOGs are formally insoluble in water. According to chemical properties, TOGs have six hydrogen bond acceptors: three oxygens in the glycerol backbone and three from each oleoyl tail (Triolein, PubChem ID: 5497163). It has been reported that TOGs are insoluble in water, but in vice versa condition, water shows some solubility with TOGs. This solubility may arise due to the presence of 6 hydrogen bond acceptors of the TOG molecule.^48^

**FIG. 3.**
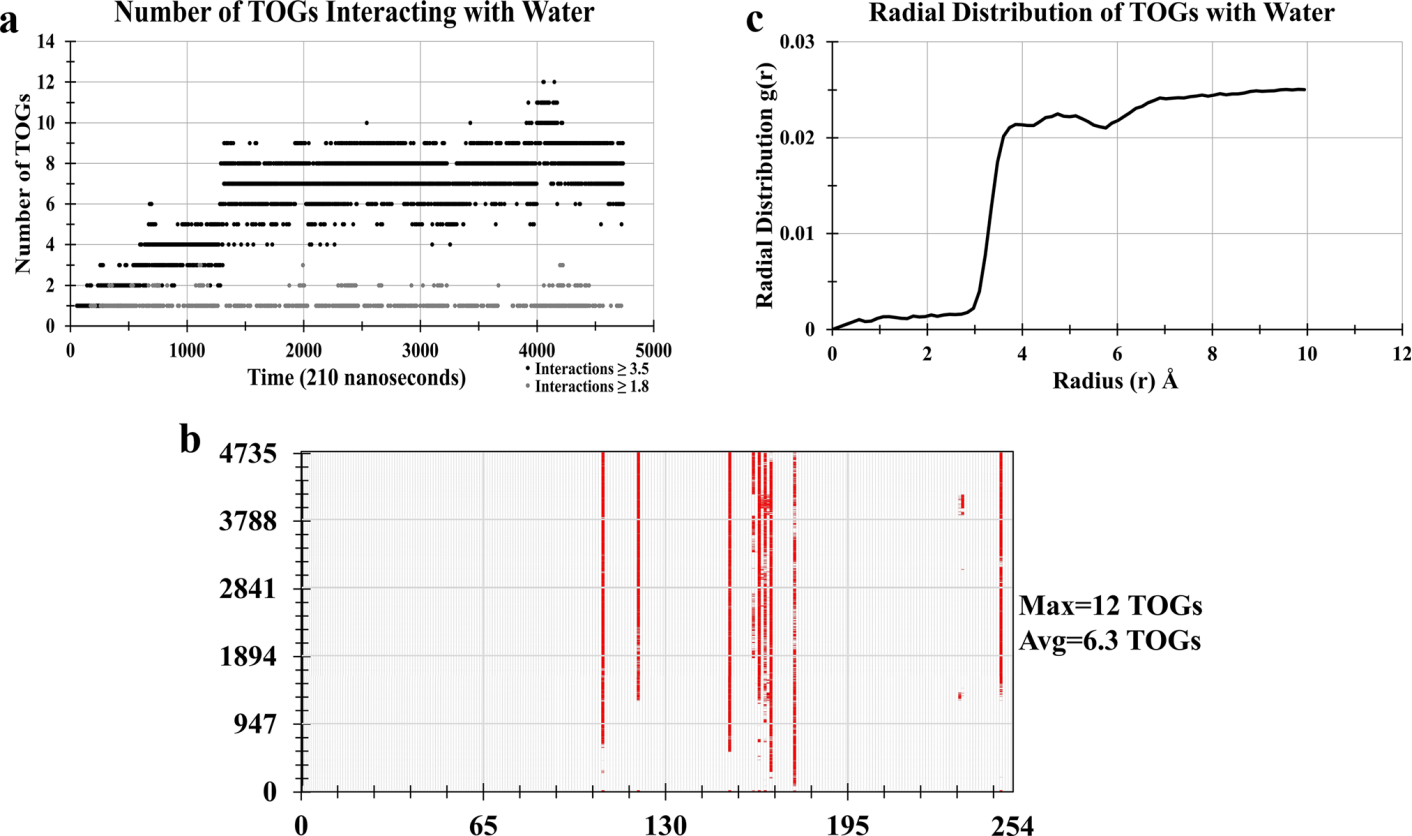
Simulation of hydrated TOG 2L system at 298 K. (a) Interaction of TOG with water performed such that any water molecule near 1.8 Å (gray dots) and 3.5 Å (black dots) were recorded. There were maximum 12 TOGs showed interactions with water at a frame while most of the time there were eight TOG interactions per frame. There were on average six TOGs showed interactions with water throughout the simulation. (b) Decomposition of TOG–water interactions for each TOG molecule. The criterion was such that if any of the oxygens of a TOG molecule shows interactions with any water molecule within 3.5 Å distance, it would show a red dot. It was observed that very few TOGs crossed TOG-water barrier to interact with water. (c) The radial distribution function (RDF) analysis with glycerol backbone carbons (C1, C2, C3) was performed with water molecules. The glycerol backbone carbons (C1, C2, C3) showed a stable interaction range of almost 3.1 Å. The values of RDF histogram values were well according to (a) data.

To observe whether our TOG system shows solubility with water as reported in previous experiments, TOGs were simulated with water. Two layers of TOGs were hydrated from top and bottom and simulated for almost 265 ns at 298 K. Analyses were performed for the last 210 ns PMD simulation. To observe TOG–water solubility, primarily any water molecule closest to oxygens of TOG (O11, O12, O21, O22, O31, O32), with a hydrogen bond distance of 1.8 Å, was identified. As shown in Fig. 3(a) (gray dots), a maximum of three TOG–water hydrogen bonds were observed throughout the simulation. The TOG–water solubility criterion was then further extended that any water molecule interacting with oxygens of TOGs within a 3.5 Å vicinity was identified [Fig. 3(a), black dots]. The interaction of each TOG molecule with water at each frame was recorded [Fig. 3(b)]. Only a few TOGs (maximum 12 TOGs) showed interactions with water. Out of these few interacting TOGs, the life of TOG–water interactions for some TOGs was transient. The TOGs whose oxygens showed interactions with water were considered soluble. TOG–water solubility was calculated as a function of the molar fraction in the solution. For conversion of the solution to the liter, the solution density was used. In our work, the solubility of TOGs in the solution was about 0.051 mol L^−1^ at 298 K. This value is near the experimental value provided in Refs. 49 and 50 at the same temperature and less than the one provided in Ref. 48. The difference in solubility values could be due to less simulation time. Still it was noticed that, even with a neutral charge, TOGs were slightly soluble with water at room temperature. It validated our TOG parameterization approach used in the current study.

Furthermore, radial distribution frequency (RDF) analysis was performed to observe the distribution of TOG molecules across water molecules [Fig. 3(c)]. The first high peak of the RDF plot was at almost 3.1 Å which is according to the TOG–water interaction data [Fig. 3(a)]. The RDF peak remained at almost the same position with short fluctuations depicting the retainment of interactions.

### Charge-corrected 4L system dismisses movement of ions/waters inside core though showed TOG micelle formation with drug molecule

Three 4L systems were prepared, one without any added explicit charges (NC). As in the real world, the A/LDs are prepared and stored in water and/or charged environments;^1^ therefore, to check the effect of ions on the charge-corrected 4L system, the second system was prepared with added balanced explicit charges on the water box (CH). In the previous 4L system, with charged TOGs, it was observed that, once inside, the Na^+^ ions started to develop interactions with the TOGs giving a view of making micelles inside. It gave rise to a fascination, i.e., what would be the behavior of charged molecules once inside the TOG core? So, to assess this fascination, in the third 4L system, a drug molecule R78 from PDB id 4I5M was introduced inside the charge-corrected 4L core (DM). R78 drug molecule has shown potent results as an inhibitor for PLK2 protein for Parkinson's disease treatment.^51^ The observations needed to make from this experiment were whether this negatively charged molecule would attract the positive ions from the surface and what would be the dynamics of the TOG core along with the drug molecule. All three systems had overall zero charge. Almost 200 ns simulation was performed for each of the charge-corrected systems, and the results were analyzed. The primary analysis of the three systems demonstrated that no positive ion disgorged across the DOPC layer toward the TOG core. Advertently, this behavior estranged former observation of the movement of positive ions toward the core from the 4L simulation of the previous study.

**FIG. 4.**
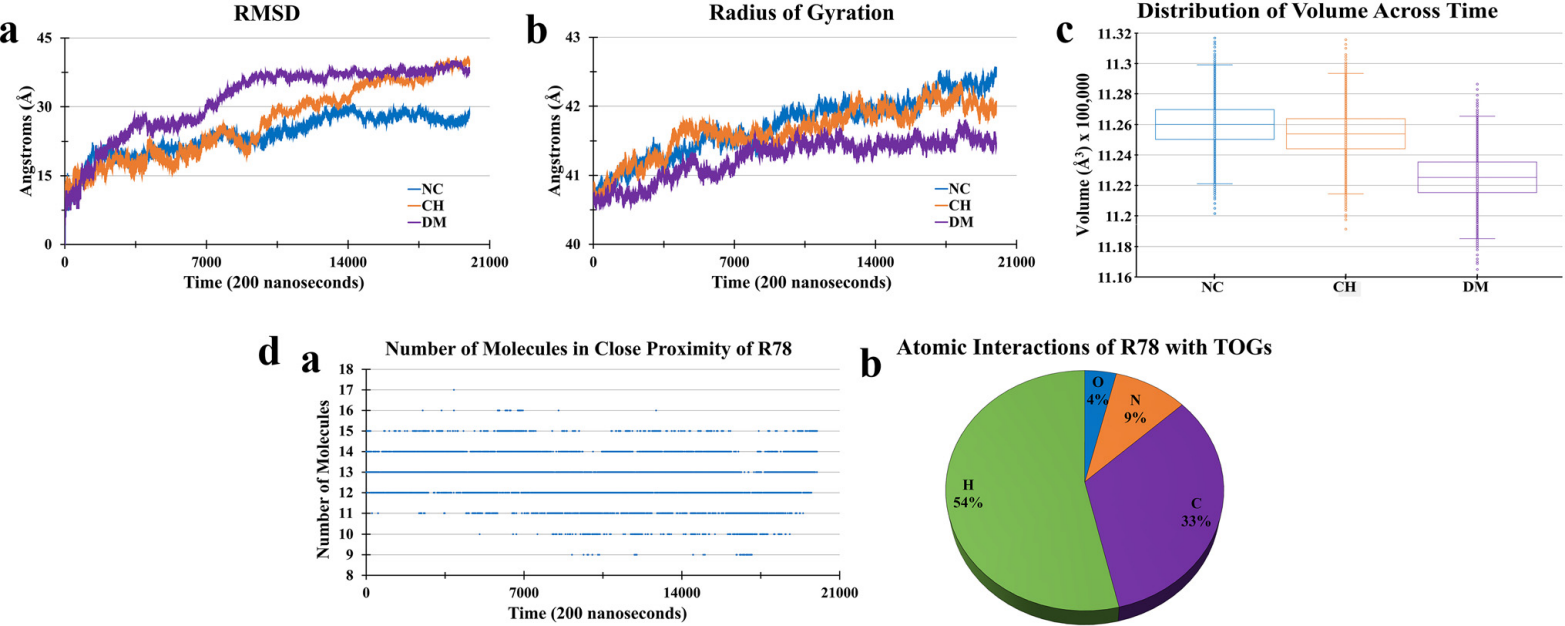
Simulation of charge-corrected adiposome mimicking 4-layer (4L) system. (a)–(c) Simulation of charge-corrected 4L system. A total three 4L systems were simulated based on presence of explicit charges (NC = no explicit charges; CH = with explicit charges; and DM = drug molecule with explicit charges). The RMSD graphs (a) demonstrated that NC and DM systems had initial RMSD peak at about 10 Å while both the systems have shown converging behavior toward the end of the simulation. In contrast, the CH system had initial RMSD peak at about 13 Å, showing increased RMSD behavior for the length of simulation. The radius of gyration (b) showed rather contrasting behavior where NC system had higher radius of gyration while CH had lower and DM systems had lowest radius of gyration. When compared with the volume of system (c), the similar behavior was observed where DM showed lowest volume as compared to CH and NC systems. (d) The number of TOG molecules (a) wrapping the drug molecule (R78) were calculated for each frame. Pie chart illustrates further R78 atomic interactions (b) with TOGs. The prevalence of atomic interactions of R78 molecule with TOGs for the total simulation time (except heating step) was 33% carbon (C), 9% nitrogen (N), 4% oxygen (O), and 54% hydrogen (H) interactions.

Furthermore, through RMSD analysis, it was observed that the NC system not only showed lower RMSD but also got converged toward the end of the simulation [Fig. 4(a)]. The CH system showed continuously increasing RMSD till the 200 ns simulation time. In comparison, the DM system showed almost similar behavior of convergence as like NC system but with initial higher RMSD. The initial higher RMSD could be caused by the interaction of drug molecule with TOGs causing substantial collisions that were stabilized later during simulation time.

The radius of gyration analysis was performed to check the behavior of the TOG layer inside the core of 4L systems. It was demonstrated that the TOGs of the NC system had the highest radius of gyration values from TOGs of CH and DM systems, while TOGs of the DM system had the lowest radius of gyration values [Fig. 4(b)]. Such behavior suggests that TOGs in the NC system had a higher degree of freedom for movement due to comparatively less environmental pressure (in this case, additional explicit ions). An analysis of the area per lipid for TOGs was performed,^52^ which suggests area per TOG for the DM system is the lowest compared to NC and CH systems (Fig. S4, Ref. 72). This behavior, in conjunction with the radius of gyration results, was further supported by the overall volume distribution of the three systems after the heating step. It was observed that the volume distribution of the NC system was higher than CH, while the CH system had higher volume distribution than the DM system. The volume distribution for the DM system was lowest [Fig. 4(c)]. One explanation for the lower volume distribution of the DM system in comparison to the other two systems could be that the R78 drug molecule may have interacted with the TOG molecules and may have made its own cluster or micelle, as was observed for Na^+^ ions in a simulation of 4L system of the previous study. Therefore, the micelle formation may have caused the TOG volume to shrink, influencing the overall volume shrinkage of the system. Additional analysis showed that, as with the first 4L system where one Na+ ion was wrapped with 3 TOG molecules at most, the R78 drug molecule in the charge-corrected simulation was wrapped with an average of 12.7 TOG molecules [Fig. 4(d-a)]. During the lifetime of an R78-TOG contact, a minimum of six TOG molecules (TOG468, TOG317, TOG290, TOG342, TOG480, and TOG469) were always in contact with R78 drug molecule for any given frame (Table II). This observation also strongly supports the formation of closed micelle within the TOG core. It answers the later fascination, i.e., the behavior of charged molecules once inside the core. The further dissection of atomic contacts of R78 with atoms of TOGs during the whole simulation run demonstrated that out of the total number of R78 atomic interactions, 4% were by Os, 9% by Ns, while Cs had 33%, and Hs were involved in 54% of total interactions [Fig. 4(d-b)]. It could be an important observation regarding addition of drug molecules to A/LDs and observing their stability dynamics. Nevertheless, for better understanding, more and different drug molecules with various drug molecule-to-TOG concentrations may provide ameliorative details about overall A/LD behavior with drugs.

**TABLE I.** Details of simulation systems for three experiments performed. Details are presented for each set of experiments performed. The components including number of atoms/molecules used with charge of system is shown here. The brief details for each experiment including remarks are also shown. For each experiment, the respective simulation parameters are shown in Table S1 in Ref. 72.

**Components**	**Number**	**Details**	**Number of atoms**	**Comments**
**E1: charge-corrected 2L system (conformation based)**				
TOG	182	91 per layer	30 394	TOG2:1 conformation
Charge of system	0		0	
**Total**	**182**		**30 394**	
TOG	288	144 per layer	48 096	TOG3 conformation
Charge of System	0		0	
**Total**	**288**	**48 096**		
**E2: charge corrected TOG 2L—water system**				
TOG	254	144 per layer	42 418	NPT ensemble
Water	12 050	∼6025 per layer	36 150	
Charge of System	0		0	
**Total**	**12 304**		**78 568**	
**E3: charge-corrected 4L system**				
**System 1 (no explicit charges)**				
DOPC	288	144 per layer	39 744	NPT ensemble (no explicit charges)
TOG	254	127 per layer	42 418	
Water	13 074	∼6537 per layer	39 222	
Charge of system	0		0	
**Total**	**13 616**		**121 384**	
**System 2 (with explicit charges)**				
DOPC	288	144 per layer	39 744	NPT ensemble (with explicit charges)
TOG	254	127 per layer	42 418	
Water	13 024	6512 per layer	39 072	
Ions	50	25 Cl^−^ 25 Na^+^	50	
Charge of system	0		0	
**Total**	**13 616**		**121 284**	
**System 3 (drug molecule with explicit charges)**				
DOPC	288	144 per layer	39 744	NPT ensemble (drug molecule with explicit charges)
TOG	252	126 per layer	42 084	
Water	13 053	6526 per layer	39 159	
Drug molecule	1		78	
Ions	21	10 Cl^−^ 11 Na^+^	21	
Charge of system	0		0	
**Total**	**13 615**		**121 086**	

**TABLE II.** R78-TOG contacts throughout simulation. There were total 23 different TOGs that interacted with R78 drug molecule at different frames. The TOGs in bold have fraction of contact as 1 suggesting they were always in contact with R78 drug molecule during complete simulation run. The contacts were calculated through “nativecontacts” command of cpptraj.

**Number**	**R78-TOG contact**	**No. of frames for a contact**	**Fraction**
**1**	**468**	**22 124**	**1**
**2**	**317**	**22 124**	**1**
**3**	**290**	**22 124**	**1**
**4**	**342**	**22 124**	**1**
**5**	**480**	**22 122**	**1**
**6**	**469**	**22 121**	**1**
7	472	22 107	0.999
8	318	22 096	0.999
9	471	22 078	0.998
10	293	21 420	0.968
11	484	21 314	0.963
12	294	20 310	0.918
13	291	18 804	0.85
14	481	11 723	0.53
15	320	8455	0.382
16	483	7997	0.361
17	319	7418	0.335
18	340	4141	0.187
19	292	2493	0.113
20	470	968	0.0438
21	314	953	0.0431
22	343	704	0.0318
23	482	353	0.016

## DISCUSSION

Computational models have been providing a good starting point to test hypotheses or conduct experiments that otherwise were not feasible.^53,54^ In several instances, the results obtained from computational models were proved experimentally when apropos techniques were available. The prominent case is the quantum calculations of Cu–Cu bonds which were subsequently validated.^55^ Therefore, the accuracy of computational models has higher significance and applications in providing effective results and advancing the field. Majorly, there are four types of simulations: QM, atomic/molecular, CG, and continuum (Fig. S1, Ref. 72). When it is about analogy and simplicity, atomic-level simulations are considered a better option due to their contiguousness with actual experiments.^28,56^ There are many methods to calculate atomic-level forcefields, but the electrostatic potential (ESP) and restrained ESP (RESP) method^57,58^ implemented by RESP-ESP charge derive (R.E.D) server^32^ is considered the best among, which provide charge reproducibility as high as 0.0001e.^59^ Due to high compatibility with AMBER, almost all AMBER forcefields utilize the RESP method with Gaussian tool.^31^ So, for this study, RESP method with Gaussian tool was used for MOG parameterization.^57^ In terms of TOG parameterization, TOG–water solubility was observed to be near experimental values, which formalizes the correct parameterization of TOGs. Even the bonds, angles, and dihedrals values of TOG parameters in this study are well-correlated with experimental crystal structure values reported in Refs. 23, 34, and 35. There were many limitations to this study: primary limitation was the computational power and available tools to prepare membrane systems. Manual membrane building was performed for some experiments due to the unavailability of tools offering the required functionality. Another limitation was related to TOG molecule building.

Quantum mechanically calculated MOG forcefield was used in this study. Charge correction was made that resulted in a neutral TOG molecule. In our results, the comparison of charge-corrected and charged 2L simulations demonstrated an almost similar type of behavior in terms of energy and other parameters [Figs. 1(a) and 1(b)]. It confirmed the previous simulation results of charged TOGs which were performed in isotropic periodic boundary conditions with uniform neutralizing plasma conditions implemented by the AMBER.^60^ Under periodic boundary conditions, the uniform neutralizing plasma considers each allocated grid as a neutral one where the grid might use smearing of net charge to calculate the relative equations including Ewald.^61–66^ In 2L simulations, larger equilibration steps were used with no observed incongruity in the system. The purpose of this study was to provide researchers with initial grounds for performing atomic-level simulations with neutral lipids, so only parameters related to overall system stability were analyzed.

TAGs are highly dynamic molecules and, in some cases, have long chains of carbon atoms. These long chains of heavy atoms increase the collision and movement rate of atoms causing TAGs to have different conformations at different timescales.^19–21^ A properly built system is of primary importance for a successful simulation and as per available literature, the current simulations have not essentially provided details of initial TAG conformation and even not TAG–TAG distances to be used for initial membrane building.^67–69^ In the current study, during the simulation run, TOG acquired multiple conformations but results demonstrated that TOG2:1 was the most populated conformation and is a more favorable choice to build an initial membrane system with 6 Å TOG–TOG distances. In the current study, the observed TOG–water solubility was 0.051 mol L^−1^ in the total miscible solution. TOG–water solubility was observed to be higher till 0.056 mol L^−1^ when solubility was observed only in water rather than the solution.

It is currently not possible to deduce whether the movement/presence of ions/molecules other than lipids in the TOG core is an artifact because there is one such example where through freeze-fracture immunocytochemistry compounded with electron microscopy, the authors showed the presence of PAT family proteins in LD core.^70^ One of the previous 4L system studies suggests that a fraction of Na^+^ ions with their water shells diffused toward the core of the 4L membrane.^30^ Few CG simulations also supported the movement of water inside the core.^13,71^ While our charge-corrected 4L simulation results ruled out the movement of Na^+^ ions with their water shells inside the core. Furthermore, a mono-negatively charged molecule was placed inside the core to check the dynamics of the 4L core. Accordingly, even the mono-charged drug molecule also did not cause movement of Na^+^ ions toward the core of 4L. Alongside it was observed that the R78 drug molecule tends to make micelle of interacting TOGs influencing the radius of gyration of TOG membrane and overall volume of the system. It is expected that this study may help researchers understand the behavior and dynamics of the neutral lipid core of A/LDs and would also help design A/LDs with explicit drug molecules.

## CONCLUSION

Lipid droplets (LDs) are a dynamic cellular organelle, whereas adiposomes are artificially produced structures mimicking LDs. The purpose of this study was to set initial grounds for the study of A/LDs at the atomic level. The results were comparable to other computational and real-world experiments. In previous studies, no clear evidence has been provided for the TOG conformation to be used for initial membrane building. This study demonstrated that TOGs prefer to stay in lamellar TOG2:1 conformation, which has also been proven in the TOG crystal structures. In terms of solubility, TOG–water solubility was about 0.051 mol L^−1^ at 298 K, well in range with experimental and computational values. In another simulation, where a drug molecule placed inside the core of adiposome mimicking four-layer system demonstrated that TOG molecules showed interactions with drug molecule, such that, to make micelle structures. These observations could be used to utilize A/LDs as efficient drug carriers. It is expected that the availability of TOG forcefield might enable future studies to be performed at the atomic level.

## Data Availability

The data related to the MOG forcefield including QM parameters used, original QM resulted molecule file, trajectories, and all other related data can be provided to researchers when requested.

## Nomenclature

A/LD: adiposome/LD
AMBER: advanced model building with energy refinements
CE: cholesteryl esters
CG: coarse-grained
CH: with explicit charges
DM: drug molecule with explicit charges
DOPC: dipalmitoyl phosphatidylcholine
ER: endoplasmic reticulum
ESP: electrostatic potential
ESP R.E.D: RESP-ESP charge derive
HDL: high density lipoprotein particles
LD: lipid droplet
MOG: 2-monooleoylglycerol
NC: no explicit charges
NPT: constant pressure and temperature
NVT: constant volume and temperature
PMD: production molecular dynamics
QM: quantum mechanical
RESP: restrained
RMSD: root mean square deviation
TAG: triacylglycerol
TOG: trioleoylglycerol
TOG2:1: two lipid tails in one direction, lipid tail opposite direction
TOG3: three lipid tails in one direction
UA: united atom
2D: 2-dimensional
2L: 2-layer/bilayer
4L: 4-layer/tetralayer
